# Real-world outcomes of mepolizumab in severe eosinophilic asthma: a retrospective cohort study

**DOI:** 10.36416/1806-3756/e20250052

**Published:** 2025-06-20

**Authors:** Pedro Fernandes, Marta Sousa, João Paulo Silva, Teresa Belo, Rita Ferro, António Reis Correia, Ana Luís Correia

**Affiliations:** 1. Centro Hospitalar Tondela-Viseu, Viseu, Portugal.

**Keywords:** Mepolizumab, Eosinophilic asthma, Severe asthma

## Abstract

**Objective:**

To evaluate the efficacy and safety of mepolizumab in patients with severe eosinophilic asthma (SEA) in a real-world clinical setting.

**Methods:**

This retrospective, observational cohort study included data from 45 SEA patients who initiated mepolizumab treatment, with 41 completing a 12-month follow-up. Demographic, clinical, and laboratory data-including exacerbation rates, forced expiratory volume in one second (FEV_1_), Asthma Control Test (ACT) and CARAT scores, and blood eosinophil counts-were extracted from patient medical records. Paired statistical tests were used to compare pre- and post-treatment outcomes, with significance set at p < 0.05.

**Results:**

Mepolizumab therapy was associated with an 81% reduction in annual exacerbation rates (from 2.68 to 0.51 events; p < 0.001) and a 21.6% improvement in FEV_1_ (from 1.71 L to 2.08 L; p < 0.001). ACT scores increased significantly from 11.91 to 21.29 (p < 0.001), with 73.2% of patients achieving well-controlled asthma (ACT ≥ 20). CARAT scores also showed significant improvement, reflecting better control of both asthma and rhinitis symptoms. Blood eosinophil counts decreased by 64% (from 525.9 to 189.4 cells/µL; p < 0.001). Overall, the treatment was well tolerated, with only one discontinuation due to a mild headache.

**Conclusion:**

In this real-world cohort, mepolizumab significantly reduced exacerbation frequency, improved lung function and symptom control, and lowered eosinophil levels over 12 months. These findings support its use as an effective and safe therapeutic option for managing severe eosinophilic asthma.

## INTRODUCTION

Severe eosinophilic asthma (SEA) is a high-burden, treatment-resistant phenotype characterized by chronic eosinophilic inflammation, frequent exacerbations, and impaired quality of life, despite optimized inhaled and systemic corticosteroid therapy. In clinical practice, real-world data have become increasingly valuable in complementing randomized controlled trials (RCTs), offering insights into the effectiveness, safety, and tolerability of treatments across broader and more diverse patient populations. For both clinicians and patients, real-world evidence supports therapeutic decision-making in routine care settings, where disease presentation, comorbidities, and treatment adherence may differ significantly from those observed in controlled trials.

Mepolizumab, a humanized monoclonal antibody targeting interleukin-5 (IL-5), represents a transformative advancement in the treatment of SEA. By selectively inhibiting IL-5, mepolizumab reduces eosinophilic activity-a key driver of inflammation in this asthma phenotype. Pivotal clinical trials, including the DREAM and MENSA studies, have demonstrated significant reductions in exacerbation rates, improvements in lung function, and enhanced asthma control with mepolizumab therapy. These findings have been further supported by real-world evidence from studies such as REDES and REALITI-A, which highlight the effectiveness and safety of mepolizumab in diverse patient populations.^1^

Despite these advances, real-world data on the impact of mepolizumab in specific populations remain limited. This retrospective, observational study evaluated the efficacy and safety of mepolizumab therapy in patients with SEA over a 12-month period, focusing on clinical outcomes such as exacerbation rates, lung function, symptom control, and blood eosinophil levels. Additionally, the study contextualizes these findings within the broader body of evidence from clinical trials and real-world studies, aiming to provide a more comprehensive understanding of mepolizumab’s role in the management of SEA.

## METHODS

This retrospective, observational cohort study was conducted in patients with SEA treated with mepolizumab. Eligible patients were identified from clinical records at a single center and included individuals aged 18 years or older who had initiated mepolizumab therapy at least 12 months prior to data collection. A documented diagnosis of SEA was required, based on established clinical criteria, including recurrent exacerbations and evidence of eosinophilic inflammation (blood eosinophil counts ≥ 150 cells/µL), in accordance with the Global Initiative for Asthma (GINA) guidelines.^10^ Patients with significant comorbid conditions that could influence SEA outcomes, such as uncontrolled sleep apnea or severe rhinitis, were noted but not excluded.

Data were retrospectively collected from electronic medical records, covering the period from January 2016 to August 2024. Extracted variables included demographic characteristics, clinical history, blood eosinophil counts, exacerbation history, lung function parameters, and Asthma Control Test (ACT) scores. Exacerbations were defined as episodes requiring systemic corticosteroids for at least three days or resulting in an emergency department visit or hospitalization. To minimize errors, the completeness and accuracy of the data were verified through cross-referencing with pharmacy records and consultation notes.

Patients received 100 mg of mepolizumab subcutaneously every four weeks, in accordance with standard clinical practice. Mepolizumab initiation was based on poor response to standard asthma therapies or persistently elevated blood eosinophil counts. Treatment adherence was monitored through regular follow-up appointments and pharmacy refill records. Concurrent asthma therapies, including inhaled corticosteroids and bronchodilators, were maintained without modification during the study period to ensure consistency in background treatment.

The primary outcome was the annualized rate of clinically significant exacerbations before and after mepolizumab initiation. Secondary outcomes included changes in lung function (measured by FEV_1_), symptom control (assessed using ACT and CARAT scores), and blood eosinophil counts. Safety outcomes, such as the incidence and severity of adverse events, were also recorded. Mild adverse events were defined as transient symptoms that did not require treatment modification, while severe adverse events necessitated treatment discontinuation or additional medical intervention. Additionally, correlations between eosinophil reduction and symptom improvement were explored.

Descriptive statistics were used to summarize baseline characteristics, including means, standard deviations, and proportions. Changes in clinical outcomes before and after treatment were analyzed using paired t-tests or Wilcoxon signed-rank tests for continuous variables, and McNemar’s test for categorical variables, depending on data distribution. Sensitivity analyses were conducted to account for variability in data collection and to confirm the robustness of the results. Statistical significance was set at p < 0.05. In order to address potential biases, subgroup analyses were performed based on age, baseline eosinophil counts, and comorbidities. All analyses were carried out using SAS software, version 9.4 (SAS Institute, Cary, NC, USA).

This study was conducted in accordance with the Declaration of Helsinki and was approved by the local ethics committee. Written informed consent was obtained from all patients when required. As a retrospective analysis, patient confidentiality was strictly maintained, and no identifiable information was included in the final dataset.

## RESULTS

### Overall baseline characteristics of the study population

A total of 45 patients with severe eosinophilic asthma (SEA) initiated treatment with mepolizumab during the study period. Of these, 41 completed the full 12-month follow-up. Four patients (8.9%) discontinued treatment prior to completion: three due to persistent symptoms despite therapy, and one due to an adverse effect (headache). It is worth noting that none of the patients who discontinued treatment achieved well-controlled asthma (ACT ≥ 20), nor did they show significant reductions in blood eosinophil counts or improvements in lung function. These findings underscore the importance of early therapeutic response assessments to guide decisions regarding treatment continuation.

Table 1 presents a comprehensive overview of the baseline demographic and clinical characteristics of the study participants. The analyzed variables included age, sex distribution, body mass index (BMI) categories, prevalence of atopy, comorbid conditions, and blood eosinophil counts. Results were presented as means with standard deviations (SD) for continuous variables and as frequencies (n) with percentages (%) for categorical variables. Notably, the study cohort exhibited substantial disease burden and clinical complexity. The group consisted of 41 individuals, predominantly female (65.9%), with a mean (SD) age of 62.7 (9.9) years. Nearly half (48.8%) of the participants were overweight, and 9.8% were classified as obese. Atopy and nasal polyposis were both prevalent, each affecting 48.8% of the cohort. 

**Table 1:** Demographic and clinical characteristics of the study population.

Variable	
Age in years, m (SD)	62.71 (9.95)
Sex	
Male, n (%)	14 (34.15)
Female, n (%)	27 (65.85)
Body mass index, mean (SD)	
Normal weight, n (%)	17 (41.46)
Overweight, n (%)	20 (48.78)
Obese, n (%)	4 (9.76)
Atopy, n (%)	
Yes	20 (48.78)
No	21 (51.22)
IgE (IU/mL); m (SD)	252.5 (303.77)
Anxiety/Depression, n (%)	
Yes	8 (19.51)
No	33 (80.49)
Sleep apnea, n (%)	
Yes	13 (31.71)
No	28 (68.29)
Rhinitis, n (%)	
Yes	30 (73.17)
No	11 (26.83)
Nasal polyps, n (%)	
Yes	20 (48.78)
No	21 (51.22)
Gastroesophageal reflux, n (%)	
Yes	4 (9.76)
No	37 (90.24)

The mean (SD) baseline blood eosinophil count of 525.94 (200.3) cells/µL highlights the eosinophilic nature of this patient population. Comorbidities were common, with anxiety or depression affecting 19.5% of patients and sleep apnea diagnosed in 31.7%. Rhinitis was particularly prevalent, present in 73.2% of the participants. These findings underscore the need for tailored interventions to address the multifaceted challenges associated with SEA.

### Exacerbations

**Figure 1:**
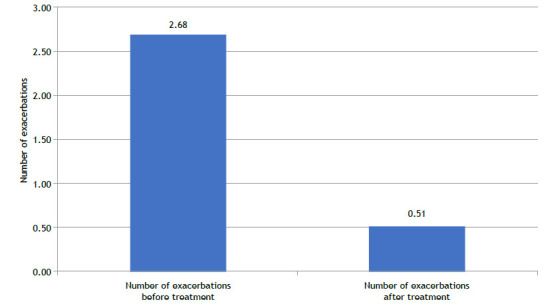
Annual Exacerbation Rates Before and After 12 Months of Mepolizumab Treatment in Patients with Severe Eosinophilic Asthma (SEA). The mean exacerbation rate decreased significantly from 2.68 (SD = 1.12) to 0.51 (SD = 0.84), representing an 81% reduction (p < 0.001), thus highlighting the substantial impact of mepolizumab in reducing disease severity and improving patient outcomes.

Mepolizumab treatment resulted in a significant reduction in asthma exacerbations. In Figure 1, it can be noted that the mean (SD) number of episodes decreased from 2.68 (1.12) in the 12 months prior to treatment to 0.51 (0.84) post-treatment, representing an 81% reduction (p < 0.001). 

### Lung function

**Figure 2:**
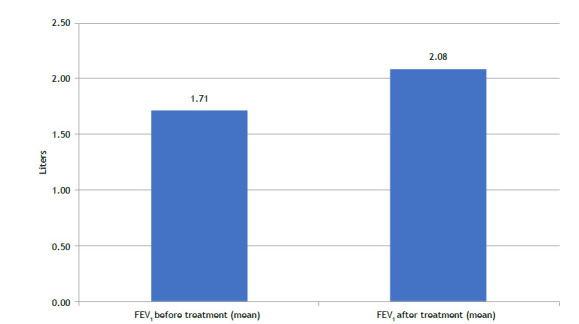
Lung Function Before and After 12 Months of Mepolizumab Treatment. This figure indicates the change in forced expiratory volume in one second (FEV_1_) among patients with severe eosinophilic asthma (SEA). The mean FEV_1_ increased significantly from 1.71 L (SD = 0.54) at baseline to 2.08 L (SD = 0.62) post-treatment, reflecting a 21.6% improvement (p < 0.001). These results underscore the efficacy of mepolizumab in enhancing lung function and reducing airway inflammation.

Significant improvements in lung function were observed following treatment with mepolizumab. The mean (SD) FEV_1_ increased from 1.71 (0.54) liters at baseline to 2.08 (0.62) liters post-treatment, with a mean absolute increase of 0.37 L (p < 0.001), corresponding to a 21.6% relative improvement (Figure 2). This outcome is consistent with findings from the MENSA study and may be partially attributed to the long-standing duration of asthma in our cohort. The average age of 62.7 years suggests the presence of potentially reversible airflow limitation, likely due to improved inflammatory control.

### Symptom control

**Figure 3:**
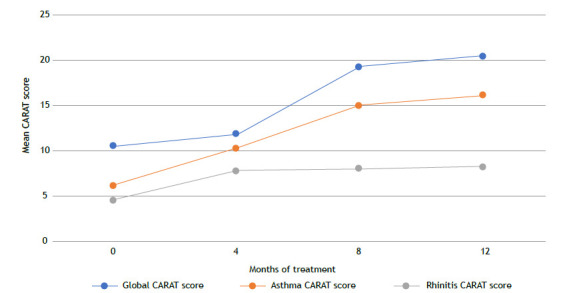
CARAT Scores Over 12 Months of Mepolizumab Treatment. This figure illustrates the progression of CARAT (Control of Allergic Rhinitis and Asthma Test) scores in patients with severe eosinophilic asthma (SEA) at baseline and after 4, 8, and 12 months of mepolizumab therapy. The mean total CARAT score improved significantly from 10.60 (SD = 4.35) at baseline to 20.54 (SD = 6.45) at 12 months (p < 0.001), indicating better control of asthma and allergic rhinitis symptoms. Subdomain scores for asthma and rhinitis also showed notable improvements over time. These results demonstrate the dual benefit of mepolizumab in managing both asthma and upper airway symptoms.

The ACT scores improved significantly, increasing from a mean (SD) of 11.91 (4.76) at baseline to 21.29 (4.79) at follow-up (p < 0.001), as shown in Figure 3. Notably, 73.2% of patients achieved scores ≥ 20, indicating well-controlled asthma, and an equal proportion met the minimal clinically important difference (MCID), with an increase of ≥3 points. At baseline, only 2.4% of patients had an ACT score ≥20, compared to 73.2% after 12 months of mepolizumab therapy. This pronounced categorical shift reflects the substantial clinical impact of mepolizumab treatment on symptom control, consistent with the significant improvement in mean ACT scores.

CARAT scores also improved significantly following treatment. At follow-up, 65.9% of patients achieved a clinically meaningful improvement, defined as an increase of ≥8 points, thus meeting the MCID. 

**Figure 4:**
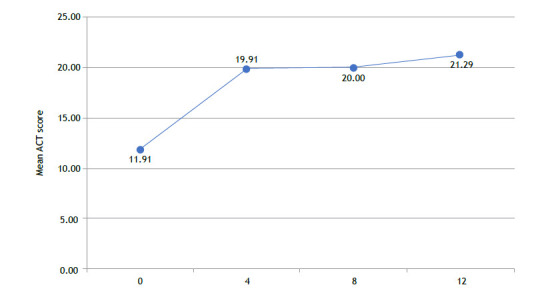
ACT Scores Over 12 Months of Mepolizumab Treatment. This figure depicts the progression of ACT (Asthma Control Test) scores in patients with severe eosinophilic asthma (SEA) at baseline and after 4, 8, and 12 months. The mean ACT score increased significantly from 11.91 (SD = 3.45) at baseline to 21.29 (SD = 3.87) at 12 months (p < 0.001), exceeding the minimal clinically important difference (MCID) of 3 points. The percentage of patients achieving well-controlled asthma (ACT ≥ 20) rose from 7.3% at baseline to 73.2% at 12 months, highlighting the substantial impact of mepolizumab on asthma symptom control.

Specifically, the CARAT subdomain scores for asthma and rhinitis demonstrated meaningful improvements. Asthma-related CARAT scores increased from a mean of 6.24 at baseline to 16.21 at 12 months, while rhinitis-related scores rose from 4.60 to 8.30 over the same period (Figure 4). These findings demonstrate the dual benefit of mepolizumab in controlling both asthma and upper airway symptoms, which are often interrelated in patients with SEA.

### Subgroup analysis based on CRSwNP and atopy status

To explore whether the clinical responses varied across phenotypic subgroups, we conducted an exploratory analysis comparing patients with and without chronic rhinosinusitis with nasal polyps (CRSwNP), as well as those classified as atopic versus non-atopic based on clinical phenotype. Patients with CRSwNP (n = 20) demonstrated numerically greater improvements in asthma control and symptom burden than those without nasal polyps, with mean ACT score increases of +11.2 vs. +8.0 and CARAT score improvements of +11.1 vs. +7.4, respectively. The reduction in the annualized exacerbation rate was also slightly greater in the CRSwNP group (-2.47 vs. -2.29). Conversely, patients with an atopic phenotype (n = 20) experienced greater reductions in exacerbations (-3.00 vs. -2.33) but showed smaller improvements in ACT (+1.0 vs. +10.8) and CARAT (+4.0 vs. +10.6) scores compared to non-atopic individuals. Given the limited sample size, these findings are descriptive and should be interpreted as hypothesis-generating.

### Blood eosinophil counts

**Figure 5:**
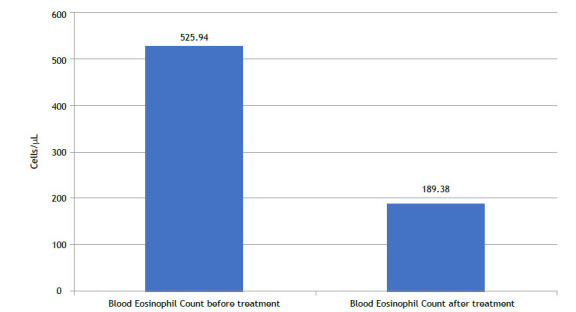
Blood Eosinophil Counts Before and After Mepolizumab Therapy. This figure indicates a significant reduction blood eosinophil counts in patients with severe eosinophilic asthma (SEA) post-treatment. The mean eosinophil count decreased from 525.9 cells/µL (SD = 200.3) at baseline to 189.4 cells/µL (SD = 75.2) post-treatment, representing a 64% reduction (p < 0.001). This decline underscores the anti-inflammatory efficacy of mepolizumab in targeting eosinophilic inflammation.

The mean (SD) blood eosinophil count at baseline was 525.94 (200.3) cells/µL, which decreased to 189.38 (122.1) cells/µL after 12 months of treatment (p < 0.001), representing a 64% reduction (Figure 5). At baseline, 12.2% of patients had eosinophil counts between 150-300 cells/µL, while 87.8% had counts above 300 cells/µL, reflecting the predominantly eosinophilic phenotype of the study cohort.

### Safety

Mepolizumab demonstrated a favorable safety profile, with only one patient reporting a mild headache that led to a switch in therapy. Transient adverse events including musculoskeletal complaints reported by only two patients, were consistent with those documented in the REDES and REALITI-A studies. Importantly, no serious adverse events were reported, and the high treatment persistence rate (97.6%) reflects the high tolerability and acceptability of mepolizumab in real-world settings.

## DISCUSSION

The findings of this study demonstrate the robust efficacy and favorable safety profile of mepolizumab in the management of severe eosinophilic asthma (SEA). The observed improvements in exacerbation rates, lung function, symptom control, and blood eosinophil counts are consistent with results from pivotal clinical trials, including DREAM and MENSA,^5^ ^,^ ^7^ as well as real-world studies such as REDES and REALITI-A. These consistencies reinforce the reliability of mepolizumab across diverse patient populations and clinical settings.

The clinical benefits observed in our study align with findings from both randomized controlled trials and real-world investigations. Notably, the 81% reduction in the annualized exacerbation rate slightly exceeds the 77.5% reduction reported in the REDES study and is greater than the 69.6% observed in REALITI-A. Similarly, our cohort showed a mean FEV_1_ increase of 0.37 L, compared to gains of approximately 0.22 L and 0.21 L in REDES and REALITI-A, respectively. Improvements in symptom control were also more pronounced, with a mean ACT score increase of 9.38 points and 73.2% of patients achieving well-controlled asthma (ACT ≥20) after 12 months-higher than the rates reported in previous real-world cohorts. Although the 64% reduction in eosinophil counts was lower than the 82.4% observed in REDES, this may reflect baseline differences in eosinophil levels and a higher prevalence of comorbidities in our cohort.

The finding that 73.2% of patients achieved well-controlled asthma (ACT ≥ 20) after 12 months reflects a robust clinical benefit, consistent with the observed improvements in exacerbation rates and lung function. Interestingly, none of the patients who discontinued therapy early demonstrated clinically meaningful improvements across these outcomes, suggesting that an early lack of response may be predictive of long-term non-responsiveness. This observation supports recent evidence advocating for a reassessment of biologic therapy efficacy within the first 4-6 months of treatment.

While consistent with previous studies, the eosinophil reduction observed in this cohort was slightly lower than the 82.4% reported in the REDES study. Potential contributing factors include differences in baseline eosinophil levels and the presence of comorbidities-particularly the high prevalence of rhinitis and sleep apnea in our cohort. Further research is warranted to investigate how these comorbidities may influence treatment outcomes.

This study’s focus on a cohort with a high burden of comorbidities-including rhinitis (73.2%) and sleep apnea (31.7%)-provides valuable insights into the effectiveness of mepolizumab in managing SEA within complex patient populations. The findings demonstrate the dual benefit of mepolizumab in addressing both lower and upper airway inflammation, which are frequently interconnected in SEA. Moreover, the significant improvement in ACT scores, with 73.2% of patients achieving well-controlled asthma (ACT ≥ 20) post-treatment, reinforces its potential to substantially improve patient quality of life.

The notable reductions in blood eosinophil counts and exacerbations reaffirm the role of IL-5 inhibition in mitigating eosinophilic inflammation. This mechanistic efficacy likely underpins the improvements in both lung function and symptom control observed in this cohort. Furthermore, the concurrent improvements in CARAT scores for both asthma- and rhinitis-related symptoms suggest that mepolizumab effectively targets systemic eosinophilic inflammation, extending its benefits beyond asthma control alone.

The observed absolute FEV_1_ gain of 370 mL over 12 months of treatment was greater than that reported in other real-world studies, such as REDES and REALITI-A, where mean improvements ranged from approximately 190 to 220 mL. One possible explanation for this more pronounced response is the longer disease duration in our cohort (mean of 22.8 years). It is conceivable that patients with longstanding asthma may have accumulated a greater eosinophilic burden and degree of airway remodeling, potentially making them more responsive to IL-5 blockade. These findings raise the hypothesis that disease chronicity may influence the magnitude of functional improvement with targeted biologic therapy, although further prospective studies are needed to confirm this association.

When considering the broader landscape of biologic therapies for severe eosinophilic asthma (SEA), mepolizumab demonstrates comparable efficacy to other IL-5-targeting agents, such as benralizumab, and biologics addressing type-2 inflammation, such as dupilumab. While mepolizumab effectively reduces eosinophil counts and exacerbation rates, real-world studies highlight its particular benefit in patients with complex comorbidities, including rhinitis and nasal polyps, where dual airway inflammation is prevalent. By inducing apoptosis of eosinophils through IL-5 receptor engagement, benralizumab may achieve faster eosinophil depletion, but it lacks extensive real-world data in diverse SEA populations. Dupilumab, which targets IL-4 and IL-13, broadens the therapeutic scope, offering significant benefits to patients with overlapping atopic conditions. The therapeutic choice should consider phenotypic differences, comorbidities, and patient preferences, such as administration frequency and delivery method. Current evidence underscores the robust efficacy of mepolizumab in SEA management; however, comparative real-world studies are necessary to further refine its positioning among biologics and optimize treatment algorithms.

The limitations of this study effectively highlight key concerns regarding its design and methodology. Its retrospective nature introduces biases, including reliance on medical records, the lack of randomization, and the absence of a control group, all of which affect generalizability and the ability to draw causal inferences. The single-center design further limits external validity, and the relatively small sample size reduces the power to detect rare adverse events or provide comprehensive safety data over extended periods.

The lack of detailed information on socioeconomic factors, adherence to concurrent therapies, and other potential confounders poses a significant challenge in fully understanding the range of variables influencing outcomes. Future prospective studies could address these limitations by enabling comprehensive data collection from the outset, incorporating randomization to minimize bias, and including control groups to strengthen causal inferences. Such an approach would also facilitate multicenter collaboration, enhancing sample diversity and statistical power, ultimately yielding more reliable and generalizable findings.

Further research should aim to address these limitations through prospective, multicenter studies with larger sample sizes. Long-term studies evaluating outcomes beyond 12 months may provide valuable insights into the durability of mepolizumab’s benefits and its impact on healthcare resource utilization. Additionally, head-to-head comparisons with other biologics targeting distinct inflammatory pathways could help refine treatment algorithms for SEA. Investigating the role of mepolizumab in specific subpopulations-such as individuals with obesity or those requiring maintenance oral corticosteroids-could further support the development of personalized treatment strategies.

In this real-world cohort, mepolizumab was associated with substantial reductions in exacerbations, marked improvements in lung function and symptom control, and a trend toward remission in selected patients-reinforcing its value in everyday clinical practice. Its favorable safety profile and high treatment persistence rates further support its suitability for long-term use, particularly in patients with significant comorbidities. These findings reinforce mepolizumab’s role as a cornerstone therapy in SEA management and highlight the need for ongoing research to optimize its use in across various clinical settings.
